# COHORT DIFFERENCES IN EARLY-LIFE SOCIOECONOMIC STATUS AND LATE-LIFE COGNITIVE IMPAIRMENT IN MEXICO

**DOI:** 10.1093/geroni/igac059.1256

**Published:** 2022-12-20

**Authors:** Brian Downer, Mariela Gutierrez, Silvia Mejia Arango, Rebeca Wong

**Affiliations:** University of Texas Medical Branch, Galveston, Texas, United States; University of Texas Medical Branch, Galveston, Texas, United States; Colegio de la Frontera Norte, Tijuana, Baja California, Mexico; University of Texas Medical Branch, Galveston, Texas, United States

## Abstract

Socioeconomic characteristics over the life course are associated with late-life cognitive impairment. However, evidence is lacking from countries like Mexico where population aging is occurring in the context of rapidly changing socioeconomic conditions. We used the Mexican Health and Aging Study to investigate differences between participants aged 60-76 in 2001 (n=5085) and 2018 (n=5947) in childhood (home with indoor toilet, parents’ education) and midlife (education, longest held occupation) socioeconomic characteristics and late life cognitive impairment. Cognitive impairment was defined as a low score on >2 out of five assessments. Most participants in the 2018 cohort lived in a home with an indoor toilet as a child (58.1%) and 36.9% had parents who both completed at least some education compared to 41.9% and 28.7% of participants in the 2001 cohort, respectively. Men and women in 2018 had on average 2.34 and 1.83 more years of education than men and women in 2001, respectively. The percentage of women with no main job and men who worked in agriculture were lower in 2018 than 2001 (women: 27.0% vs. 34.6%; men: 23.3% vs. 30.4%). The 2018 cohort had lower odds for cognitive impairment when adjusting for age, sex, marital status, and living in a rural/urban community (OR=0.67 95% CI=0.56-0.81). This difference was reduced after adjusting for childhood socioeconomic measures (OR=0.76 95% CI=0.67-0.86) and was no longer statistically significant after adding midlife socioeconomic measures (OR=0.98 95% CI=0.86-1.12). These findings suggest that improved early-life socioeconomic conditions in Mexico contribute to birth-cohort differences in late-life cognitive impairment.

